# Ultrahigh-field MRI: where it really makes a difference

**DOI:** 10.1007/s00117-023-01184-x

**Published:** 2023-08-16

**Authors:** Siegfried Trattnig, Gilbert Hangel, Simon D. Robinson, Vladimir Juras, Pavol Szomolanyi, Assunta Dal-Bianco

**Affiliations:** 1 High-Field MR Center – 7T MR, Department of Biomedical Imaging and Image-guided Therapy, Medical University Vienna, Lazarettgasse 14, 1090 Vienna, Austria; 2 Department of Neurology, Medical University of Vienna, Währinger Gürtel 18–20, 1090 Vienna, Austria; 3 Department of Imaging Methods, Institute of Measurement Science, Slovak Academy of Sciences, Dubravska cesta 9, 84104 Bratislava, Slovakia; 4 Medical University of Vienna, Comprehensive Center for Clinical Neurosciences & Mental Health, Vienna, Austria

**Keywords:** 7 Tesla, Multiple sclerosis, Spectroscopic imaging, Susceptibility weighted imaging, Sodium imaging, 7 Tesla, Multiple Sklerosa, Spektroskopische Bildgebung, Suzeptibilitätsgewichtete Bildgebung, Natriumbildgebung

## Abstract

**Background:**

Currently, two major magnetic resonance (MR) vendors provide commercial 7‑T scanners that are approved by the Food and Drug Administration (FDA) for clinical application. There is growing interest in ultrahigh-field MRI because of the improved clinical results in terms of morphological detail, as well as functional and metabolic imaging capabilities.

**Materials and methods:**

The 7‑T systems benefit from a higher signal-to-noise ratio, which scales supralinearly with field strength, a supralinear increase in the blood oxygenation level dependent (BOLD) contrast for functional MRI and susceptibility weighted imaging (SWI), and the chemical shift increases linearly with field strength with consequently higher spectral resolution.

**Results:**

In multiple sclerosis (MS), 7‑T imaging enables visualization of cortical lesions, the central vein sign, and paramagnetic rim lesions, which may be beneficial for the differential diagnosis between MS and other neuroinflammatory diseases in challenging and inconclusive clinical presentations and are seen as promising biomarkers for prognosis and treatment monitoring. The recent development of high-resolution proton MR spectroscopic imaging in clinically reasonable scan times has provided new insights into tumor metabolism and tumor grading as well as into early metabolic changes that may precede inflammatory processes in MS. This technique also improves the detection of epileptogenic foci in the brain. Multi-nuclear clinical applications, such as sodium imaging, have shown great potential for the evaluation of repair tissue quality after cartilage transplantation and in the monitoring of newly developed cartilage regenerative drugs for osteoarthritis.

**Conclusion:**

For special clinical applications, such as SWI in MS, MR spectroscopic imaging in tumors, MS and epilepsy, and sodium imaging in cartilage repair, 7T may become a new standard.

## Abstract (de)

**Hintergrund:**

Derzeit bieten 2 Hersteller von Geräten für die Magnetresonanztomographie (MRT) kommerzielle 7‑T-MRT-Geräte mit Zulassung der US-amerikanischen Food and Drug Administration (FDA) für die klinische Anwendung an. Es besteht ein zunehmendes Interesse an Ultrahochfeld-MRT sowohl aufgrund verbesserter klinischer Ergebnisse bei morphologischen Details als auch bei den funktionellen und metabolischen bildgebenden Möglichkeiten.

**Material und Methoden:**

Vorteile der 7‑T-Technik sind ein höheres Signal-zu-Rausch-Verhältnis, das von 3 T auf 7 T sogar supralinear zunimmt, eine ebenfalls supralineare Zunahme des Blood-Oxygenation-Level-Dependent(BOLD)-Kontrasts für funktionelle MRT und die suszeptibilitätsgewichtete Bildgebung sowie die lineare Zunahme des Chemical Shift mit konsekutiv höherer spektraler Auflösung.

**Ergebnisse:**

Bei multipler Sklerose (MS) ermöglicht die 7‑T-Technik die Visualisierung von kortikalen Läsionen, das zentrale Venenzeichen und den Nachweis von paramagnetischen Randsaumläsionen, die für die Differenzialdiagnose zwischen MS und anderen neuroinflammatorischen Erkrankungen bei schwierigen, unklaren klinischen Symptomen von Vorteil sein können sowie als vielversprechende Biomarker für Prognose- und Therapiemonitoring gesehen werden. Die kürzlich entwickelte hochauflösende Protonen-MR-Spektroskopie in klinisch noch vertretbaren Untersuchungszeiten liefert neue Einsichten in den Tumorstoffwechsel und das Grading von Tumoren, in frühe metabolische Veränderungen, die entzündlichen Prozessen bei MS vorausgehen können, und verbessert den Nachweis von epileptogenen Herden im Gehirn. Klinische Multi-Kern-Anwendungen, wie beispielsweise die Natriumbildgebung, weisen ein hohes Potenzial für die Evaluation der Qualität des Ersatzgewebes nach Knorpeltransplantationen und im Monitoring von neu entwickelten knorpelregenerativen Medikamenten zur Behandlung der Gelenkarthrose auf.

**Schlussfolgerung:**

Für besondere klinische Anwendungen wie z. B. SWI bei MS, MR-spektroskopische Bildgebung beim Hirntumorgrading und Monitoring unter Therapie oder beim Nachweis von epileptischen Foci, und der Anwendung von Multikern-MR wie die Natriumbildgebung zur Qualitätsbeurteilung von Knorpelersatztherapien, hat 7T das Potenzial ein neuer bildgebender Standard zu werden.

## Brief introduction of the subject

Three major MR vendors provide commercial 7‑T units for clinical research under ethical permission and two of these have Food and Drug Administration approval for clinical application; the number of operating 7‑T systems has increased to over 100 in recent years. This increase indicates the growing interest in ultrahigh-field (UHF) MRI because of the improved clinical results with regard to morphological detail, as well as functional and metabolic imaging capabilities.

The purpose of this article is to provide an overview of the clinical applications in which 7‑T imaging would be a possible game-changer and may become the new standard in specific imaging indications.

## Magnetic resonance spectroscopic imaging at 7T and its properties

Beyond 7‑T signal-to-noise ratio (SNR) gains, MRSI profits mainly from the increased spectral resolution of molecular MR resonances, which enables an easier quantification of overlapping resonances [1]. Examples of this are the neurotransmitter glutamate, the amino acids glutamine (Gln), and glycine (Gly) [2], or the difficult-to-quantify oncometabolite 2‑hydroxyglut-rate (2HG; [3]). At the same time, UHF SNR can be leveraged to increase spatial resolution, enabling the resolution of metabolic variation in the healthy brain as well as in pathologies ranging from brain tumors to multiple sclerosis (MS; [1]). These advantages, however, are accompanied by decreased B_0_ and B_1_ homogeneity, stronger chemical shift displacement errors, and shorter T_2_ relaxation times, which, among other issues, can lead to aggravated lipid contamination and make techniques that are in widespread use at lower field strengths, such as point-resolved spectroscopy (PRESS), ineffective [1]. Yet, the unavailability of advanced MRSI sequences that could address these challenges for clinical users limit the wider use of 7‑T MRSI.

## New 7-T MRSI methods

Approaches optimized for 7 T generally maintain either excitation or inversion stability by using adiabatic pulses [1] resistant to B_1_ inhomogeneity, in the former case, or direct free induction decay (FID) acquisition that is resistant to flip angle variations, in the latter case [4]. As with the most common fast MRSI technique at 3 T, echo-planar spectroscopic imaging (EPSI) is difficult to adapt to 7 T spectral bandwidth requirements and, therefore, can acquire only high-resolution neurochemical maps with long scan times and few slices. Other advanced trajectories, such as concentric circles, have been demonstrated as means by which to enable high-resolution MRSI within minutes [4]. The combination of FID acquisition and concentric circular readout has been shown for the 3D-MRSI acquisition of the human cerebrum at 3.4-mm isometric resolution within 15 min [4]. More specialized MRI methods, such as those for 2HG quantification, have also been published [3]. Still, there is a lack of end-user-friendly postprocessing pipeline, although the international MRSI community has recently established new consensus standards.

## Examples of new frontiers for 7-T MRSI applications

### MRSI for brain tumors

**Fig. 1:**
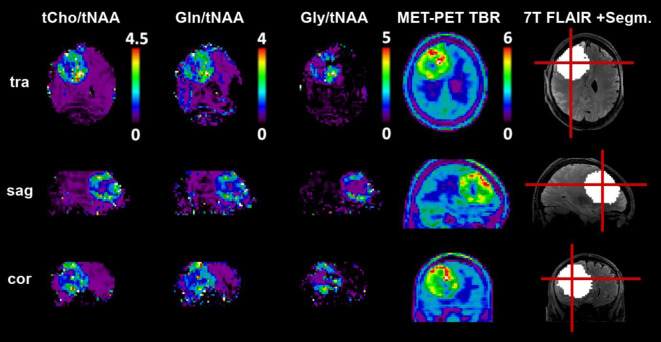
High-resolution 7‑T MRSI-derived maps of glutamine and glycine compared to MET-PET in a patient with astrocytoma grade 4 (WHO 2021) show high correspondence. Although different amino acids are used to investigate tumor metabolism, a similar spheroid hotspot is discernible for all. Adapted from [5] under Creative Commons License

The potential of 7‑T MRSI has been shown for different pathologies, such as the ability to image metabolic deviations of small MS lesions thanks to resolutions below 3 mm [1], but most published studies have focused on the spatial and metabolic resolution of brain tumors. Especially in diffuse gliomas, the possibility to better define infiltration is a noteworthy goal from a neurosurgical perspective; however, the majority of research has focused on the investigation of oncometabolites (2HG, Gln, Gly), which could enable noninvasive determination of tumor molecular pathologies, such as IDH mutation status [2]. Recently, research has demonstrated that high-resolution images of Gln and Gly, both amino acids, correspond better to amino acid-PET than the clinical marker of reference, total choline [5], as demonstrated in Fig. 1.

### MRSI for epilepsy

**Fig. 2:**
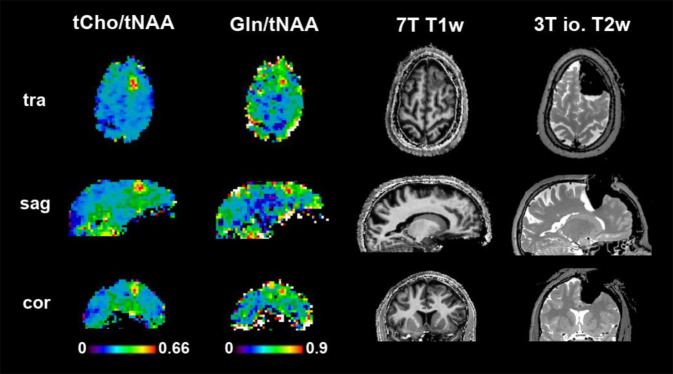
High-resolution 7‑T MRSI-derived maps of choline and glutamine in a patient with focal cortical dysplasia that was histopathologically confirmed as type 2b. These metabolic changes align well with structural findings and the extent of resection that was defined using clinical MRI. In follow-ups, the patient was confirmed to have a class 1 outcome according to ILAE, i.e., complete seizure freedom. Preliminary data from the High-Field MR Centre of the Medical University of Vienna

The range of pathologies that cause epilepsy is another theoretical application of interest for MRSI, but as drug-resistant epilepsy often is not clearly delineated on structural MRI or is not outright MR-negative, this makes the placement of MRSI volumes challenging. Despite these hurdles, initial 7‑T MRSI studies have found the same metabolic trends known from lower fields, i.e., decreased tNAA/tCr and increased tCho/tNAA [6] and have even imaged Glu and gamma-aminobutyric acid [7]. Initial results for high-resolution 7‑T 3D FID-MRSI (Fig. 2) are promising, but require a more thorough analysis before any definitive conclusions can be drawn. Compared to lower fields, 7‑T MRSI could be a game-changer as the higher spatial and spectral resolution should enable a better spatial and neurochemical delineation of epileptogenic lesions or networks.

### MRSI for multiple sclerosis

**Fig. 3:**
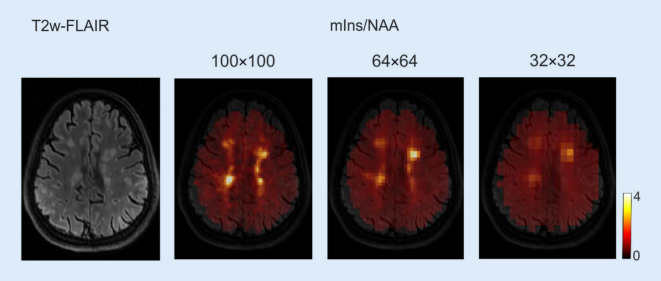
Ultrahigh-resolution, single-slice MRSI at 7 T, in this case with a 100 × 100 matrix corresponding to 2.2 × 2.2 mm^2^ in-plane size enables the detection of small multiple sclerosis lesions with greater detail than with resolutions previously available to research

Due to the smaller size of lesions in MS compared to tumors, increased MRSI resolution is desirable for improved lesion detection (Fig. 3; [8]). With ultrahigh-resolution MRSI, larger areas of neurochemical alterations become visible. The most relevant of these changes are a decrease of NAA from neuroaxonal damage and an increase of myo-Inositol (mIns) from gliosis and neuroinflammation. Mapping such changes in inflammation could be developed into a marker for disease progression, as a correlation of mIns and NAA changes with the EDSS disability score early in the course of disease was recently demonstrated [9].

## Ultrahigh-field MRI for multiple sclerosis

Susceptibility imaging encompasses MRI methods that are based on the effects that the magnetic susceptibility of tissue has upon the static magnetic field. The modified field is reflected in both how rapidly the magnitude of the gradient-echo signal decays (which is characterized by T_2_*) and through the local phase deviations in the static magnetic field B_0_. A field map can be used to calculate the underlying magnetic susceptibility in quantitative susceptibility mapping [10]. Alternatively, filtered phase and magnitude images can be combined to generate vessel contrast in susceptibility weighted images (SWI).

Susceptibility-based methods benefit particularly from 7 T due to the sensitivity of gradient-echo images to high-field [11] and UHF [12], as the susceptibility-induced variations in the static magnetic field, ∆B_0_, increase in proportion to the strength of the field itself. Recently, SWI has become something of a poster child for UHF, with 7‑T images showing an exquisite and much more complete representation of the cerebral vascular architecture than 3‑T images.

Furthermore, SWI has found the most widespread clinical adoption, with applications encompassing stroke, tumor imaging, and a wide range of pathologies with a neuroinflammatory or neurodegenerative component. In particular, MS imaging has significantly improved.

### Vessel and iron visualization in MS

The central vein in MS lesions, the “central vein sign” (CVS), is a highly specific pathologic hallmark in MS. Due to technological improvements, the threshold of 40% of lesions with CVS can now be used to distinguish MS from MS-like diseases in clinically inconclusive cases [13].

Furthermore, visualization of iron in MS has gained clinical relevance over the past decade.

**Fig. 4:**
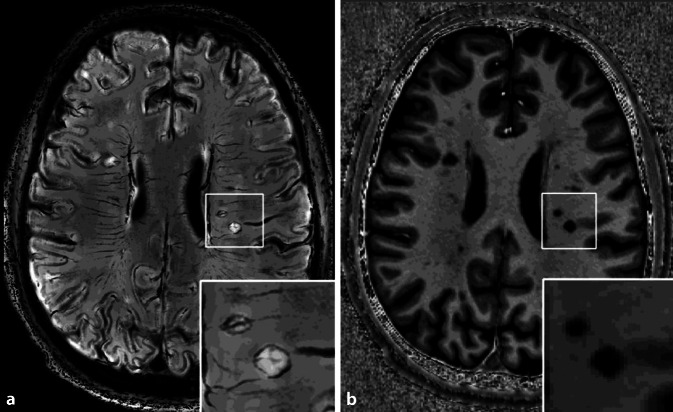
7‑T FLAIR-SWI (**a**) and MP2RAGE (**b**) images show cerebral white matter lesions in a 38-year-old male patient with relapsing-remitting multiple sclerosis with a disease duration of 3 years, an Expanded Disability Status Scale of 2.5, and a Multiple Sclerosis Severity Score of 6. Image **a** shows FLAIR-hyperintense lesions with (paramagnetic rim lesions [PRLs]s) and without (non-PRLs) SWI-hypointense rims. Corresponding MP2RAGE lesions (**b**) show increased T1-hypointensity in PRLs compared to non-PRLs, indicating the prominent tissue destruction of PRLs. Two PRLs with a central vein sign are magnified in the white rectangle in (**a**) and (**b**)

The main sources of iron accumulation in the central nervous system are erythrocytes leaking through the blood–brain barrier as well as the degradation of iron-containing oligodendrocytes and myelin [14, 15]. To protect against cytotoxic iron-induced brain tissue damage [16], microglial cells and macrophages phagocytose the free redox-active iron accumulations in the lesion center and over time form a proinflammatory rim at the lesion borders [14] from where slow lesion expansion proceeds. The iron rim eventually leads to marked tissue destruction in and around these lesions [14, 17, 18] and can be visualized due to the iron-laden microglia cells and macrophages on MRI, enabling the detection of slowly expanding iron rim lesions (IRLs), also called “paramagnetic rim lesions” (PRLs), which indicate chronic activity. The IRLs/PRLs are clinically relevant as they lead to pronounced intra- and perilesional brain tissue destruction and to a worse clinical disability with earlier conversion to progression [14, 18]. Therefore, IRLs are a promising biomarker for progressive MS. To date, the following MRI sequences have been used to visualize veins and iron at both 3‑T and 7‑T MRI: phase; SWI; quantitative susceptibility mapping; FLAIR*; R_2_*; and T_2_* [19]. For even greater optimized visualization, the so-called contrast FLAIR-SWI, a postprocessing fusion technique of 3‑T FLAIR and 7‑T SWI, was proposed by Grabner et al. in 2011, which registered the FLAIR data (3-T) to the 7‑T SWI phase data and used these FLAIR data rather than SWI magnitude data. This technique was performed successfully for patients with MS and, since then, has offered new insights into the pathogenesis of MS lesions [20]. Imaging of lesions in FLAIR-SWI, therefore, combines the radiologically known hyperintense MS lesions with overlying high-resolution visualization of iron depositions and veins (Fig. 4).

### Differential diagnosis for other diseases

As central plaque veins are highly specific for MS, their presence may be helpful in distinguishing MS from non-MS patients [13]. 7‑T imaging was more advantageous in visualizing central veins, as these vessels could be found in 87% of MS lesions using 7‑T and only 45% using 3‑T T_2_* imaging [21].

Using the central vein sign, MS could be differentiated from neuromyelitis optica spectrum disorders (NMOSDs) and Susac syndrome [22]. Furthermore, the literature indicates that IRLs/PRLs are a characteristic MS feature [23]. Jang et al. [23] found PRLs in 26 of 32 patients with MS (81.2%) and in only one of 21 patients with NMOSD (4.8%). Another recent international, multicenter, clinical research-based study of 438 patients found that IRLs were significantly more frequent in MS compared to other neurologic inflammatory, infectious, and noninflammatory diseases (52% MS vs. 7% non-MS), yielding high specificity (93%) in distinguishing MS from non-MS [24].

### Cortical lesions

**Fig. 5:**
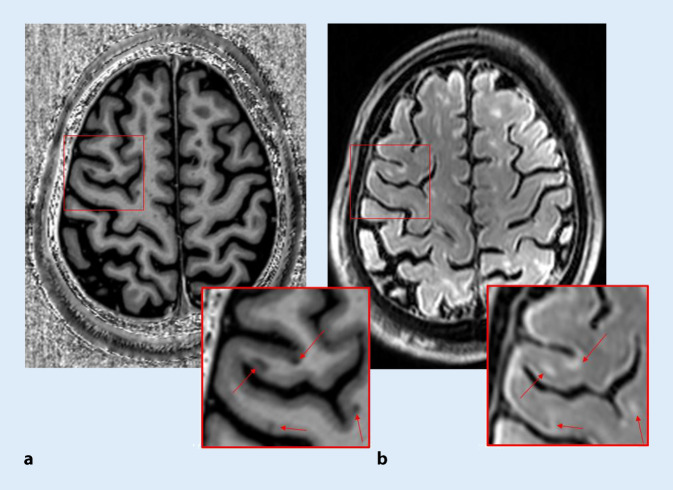
A number of cortical lesions in a patient with multiple sclerosis (39 years old, male) are well visualized on 7‑T MP2RAGE (**a**) and FLAIR (**b**)

The detection of cortical lesions benefits from higher field strengths (7 T) in terms of increased spatial resolution and increased MR contrast to the adjacent and only sparsely myelinated normal-appearing gray matter, particularly for the detection of small cortical lesions. The most useful sequences for cortical lesions are T_2_*, MP2RAGE, 3D-FLAIR, and DIR. Several studies have been published dealing with the question of whether more gray matter lesions or cortical lesions can be detected using UHF MRI compared to the clinical standard at 3 T. On average, 7‑T sequences detect 52% more cortical lesions than the best-performing 3‑T sequence. In particular, subpial and intracortical lesions are better detected at 7 T than at 3 T ([25]; Fig. 5). However, white matter (WM) lesion detection was not increased at 7 T compared to 3 T. Given the clinical relevance of GM abnormalities, this may have consequences for clinical outcome measures, prognostic classification, and future diagnostic criteria. Cortical lesions are prevalent in MS, but are poorly detected using MRI.

## Sodium (_23_Na) imaging of cartilage repair tissue

The goal of cartilage repair surgery techniques is to restore the cartilage surface and function, to allow pain-free motion of the joint, and to prevent further cartilage degeneration by providing cartilage repair tissue that has the same composition, structure, and mechanical properties as native articular cartilage. Articular cartilage defects are currently treated with a number of different surgical interventions, such as bone marrow stimulating techniques, auto-/allografting techniques, and advanced cell-based repair techniques.

Advanced cell-based repair techniques, such as autologous chondrocyte implantation (ACI), use donor-derived chondrocytes (mostly autografts) to reconstruct the cartilage defect. Autologous chondrocyte implantation was the first cell engineering approach to the treatment of cartilage lesions [26]. Although ACI can produce a hyaline-like repair tissue in some specimens, this tissue is not histochemically or morphologically identical to hyaline cartilage and fibrocartilage was found in some of the samples [27]. Although there were no significant differences in the histological quality of repair tissue between patients after ACI and microfracturing (MFX) 2 years after surgery, ACI patients more often showed hyaline-like repair tissue than MFX patients [28].

In addition to morphological MRI of cartilage repair tissue, an advanced method with which to nondestructively and quantitatively monitor parameters that reflect the biochemical status of cartilage repair tissue is a necessity for studies that seek to elucidate the natural maturation of matrix-associated ACI (MACI) grafts and the efficacy of the technique, and to answer the question of whether hyaline-like cartilage tissue really develops. For example, glycosaminoglycans (GAG) are known to be responsible for biomechanical properties such as the stiffness of cartilage, which gains even more importance with cartilage implants, and the content and organization of the collagen network reflects further mechanical properties of cartilage.

For many years, delayed gadolinium-enhanced MRI of cartilage (dGEMRIC) has been the method of choice for the evaluation of the GAG content in articular cartilage [29]. However, this method suffers from several drawbacks that distract from its clinical application, such as the application of a bolus of 0.2 mmol contrast agent per kilogram body weight (double dose), which, with the risk for nephrogenic systemic fibrosis, is problematic. Moreover, the detection of gadolinium depositions in the brain have finally led to the removal from the European market of all linear gadolinium contrast agents, including Magnevist, which has thus far been used for dGEMRIC. At 90 min after contrast application, MRI should be performed, which is difficult to handle in clinical routine examinations and, in addition, the delay differs from region to region within one joint and between joints.

Therefore, there is a need for non-contrast MR methods that could be GAG-specific and avoid all problems associated with dGEMRIC.

Proteoglycans are key macromolecules in various connective tissues, such as cartilage, tendons, or intervertebral discs. They contain sulfated GAG side chains that consist of a high concentration of negatively charged sulfate and carboxyl groups, and thus provide negative fixed charge density to the connective tissues, which is responsible for important physical properties of proteoglycans. The negative fixed charge density attracts positively charged ions (mainly sodium), and thus sodium ions are in balance with the proteoglycan content. It has been shown that the negative fixed charge density of cartilage correlates with the GAG concentration of cartilage [30].

**Fig. 6:**
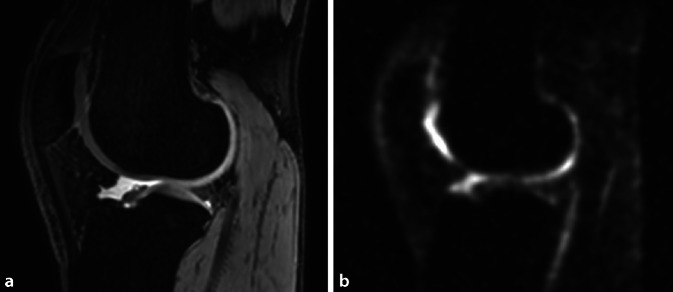
An image of the right knee of a 30-year-old male patient in a sagittal orientation generated with **a** proton (^1^H) MRI three-dimensional double-echo steady-state sequence (DESS) with an in-plane resolution of (0.5 mm)^2^ and, **b** sodium (^23^Na) three-dimensional density adapted radial projection (DA-3DPR) sequence with an isotropic resolution of (2.0 mm)^3^. The images demonstrate high morphological similarity; however, ^23^Na-MRI might provide additional information on the biochemical state of the cartilage tissue

Thus, sodium MRI can be useful for the direct and noninvasive evaluation of the GAG content in cartilage, tendons, and intervertebral discs. Unfortunately, this method is also challenging. Sodium MR sensitivity is only 9% of that of proton MR sensitivity, and the sodium in vivo concentration is several thousand times lower than the proton concentration (depending on the type of human tissue). As a consequence, the SNR of sodium MRI is 3000 to 20,000 times lower (depending on the organ) compared to the SNR of typical proton MRI. Moreover, sodium in biological tissues exhibits very short bi-exponential transversal relaxation T_2_ times, which require sequences able to acquire MR signal at very short echo times (under 2–3 ms). As a result of these factors, sodium images are acquired with a lower SNR (10–40), lower resolution (2–5 mm), and longer measurement times (10–30 min) than proton images (Fig. 6).

Several studies have used sodium MRI to evaluate tissue after cartilage repair surgery. The first sodium MR images of patients after MACI cartilage repair were published in 2010 [31]. The sodium-normalized values were significantly lower in repair tissue than in normal-appearing reference cartilage. In another study [32], sodium MRI at 7 T was used to evaluate repair tissue after two different types of cartilage repair techniques: bone marrow stimulation (BMS) techniques (Pridie drilling and MFX) and MACI. For a more accurate comparison between repair techniques, each MACI patient was matched with one BMS patient according to age, postoperative interval, and defect location. The main finding of this study was that sodium-normalized values were significantly higher in MACI than in BMS repair tissue. Nevertheless, the morphological appearance of the repair tissue, evaluated by the MOCART scoring system, was not different between BMS and MACI patients. The results suggest a higher GAG content, and therefore repair tissue of higher quality, in MACI than in BMS patients. Sodium imaging can distinguish between repair tissues with different GAG content, and thus can be useful for the noninvasive evaluation of the performance of new cartilage repair techniques. Due to the low resolution of sodium images, partial volume effects from surrounding tissues must be corrected. To minimize contamination from synovial fluid, fluid-suppression techniques should be employed [33]. Meanwhile, sodium imaging as a parameter for cartilage quality has been used in proof-of-concept studies for clinical trials to evaluate the efficacy of newly developed cartilage regenerative drugs for patients with osteoarthritis.

## Practical conclusion

- 7 T benefits from a higher signal-to-noise ratio, increased BOLD contrast, higher spectral resolution, and the possibility of X‑nuclei imaging in clinically reasonable measurement times.
- These technical benefits of 7 T can be used clinically in multiple sclerosis (MS) to visualize cortical lesions, the central vein sign, and paramagnetic rim lesions and may be used as a potential biomarker for prognosis and treatment monitoring in future.
- High-resolution proton magnetic resonance spectroscopic imaging in clinically reasonable scan times is a powerful tool for visualizing abnormal metabolism in tumors for better grading and optimal biopsy localization as well as metabolic changes that may precede inflammatory processes in MS, and for improving the detection of epileptogenic foci in the brain. X‑nuclear clinical applications, such as sodium imaging, have shown great potential for the evaluation of repair tissue quality after different cartilage repair surgeries and newly developed cartilage regenerative drugs for osteoarthritis.

## Abbreviations

ACI: Autologous chondrocyte implantation
BMS: Bone marrow stimulation
BOLD: Blood oxygenation level dependent
CVS: Central vein sign
DIR: Double inversion recovery
dGEMRIC: Delayed gadolinium-enhanced MRI of cartilage
EDSS: Expanded Disability Status Scale
EPSI: Echo-planar spectroscopic imaging
FID: Free induction decay
FLAIR: Fluid attenuated inversion recovery
GAG: Glycosaminoglycan
Gln: Glutamine
Gly: Glycine
GM: Gray matter
2HG: 2‑hydroxyglutarate
IRL: Iron rim lesion
IVD: Intervertebral disc
MACI: Matrix-associated autologous chondrocyte implantation
MFX: Microfracturing
mIns: Myo-Inositol
MOCART: Magnetic Resonance Observation of Cartilage Repair Tissue
MP2RAGE: Two echoes variant of magnetization prepared rapid gradient echo
MR: Magnetic resonance
MRSI: Magnetic resonance spectroscopic imaging
MS: Multiple sclerosis
NMOSD: Neuromyelitis optica spectrum disorders
PRESS: Point-resolved spectroscopy
PRL: Paramagnetic rim lesion
SNR: Signal-to-noise ratio
SWI: Susceptibility weighted imaging
tNAA: Total *N*-acetylaspartate
UHF: Ultrahigh-field
WM: White matter

## Declarations

For this article no studies with human participants or animals were performed by any of the authors. All studies mentioned were in accordance with the ethical standards indicated in each case.

The supplement containing this article is not sponsored by industry.
