# Erratum to: Ultrahigh-field MRI: where it really makes a difference

**DOI:** 10.1007/s00117-024-01281-5

**Published:** 2024-02-23

**Authors:** Siegfried Trattnig, Gilbert Hangel, Simon D. Robinson, Vladimir Juras, Pavol Szomolanyi, Assunta Dal-Bianco

**Affiliations:** 1 High-Field MR Center – 7T MR, Department of Biomedical Imaging and Image-guided Therapy, Medical University Vienna, Lazarettgasse 14, 1090 Vienna, Austria; 2 Department of Neurology, Medical University of Vienna, Währinger Gürtel 18–20, 1090 Vienna, Austria; 3 Department of Imaging Methods, Institute of Measurement Science, Slovak Academy of Sciences, Dubravska cesta 9, 84104 Bratislava, Slovakia; 4 Medical University of Vienna, Comprehensive Center for Clinical Neurosciences & Mental Health, Vienna, Austria

**Erratum to:**

**Radiologie 2023**

10.1007/s00117-023-01184-x

We acknowledge that we have taken some text passages from the section “Sodium(23Na) imaging of cartilage repair tissue” of this review article [1] from previous publications by the same author(s) [2–4]. We apologise for the inappropriate overlap between our own publications which was related to original text material for a large project application in the past which was later used for publications of our group. However, there is still sufficient new material and literature in the article to justify its publication.
